# Case Report: Two cases of recurrences at the suprasternal space and lymph nodes between the sternocleidomastoid and sternohyoid muscles in papillary thyroid carcinoma

**DOI:** 10.3389/fsurg.2023.1258259

**Published:** 2024-01-04

**Authors:** Hae Won Choi, Chang Myeon Song, Yong Bae Ji, Kyung Tae

**Affiliations:** Department of Otorhinolaryngology-Head and Neck Surgery, College of Medicine, Hanyang University, Seoul, Republic of Korea

**Keywords:** papillary thyroid carcinoma, lymph node metastasis, central neck dissection, suprasternal space, recurrence, thyroid cancer

## Abstract

Recently, lymph node metastasis to the suprasternal space (SSLN) and lymph nodes between the sternocleidomastoid and sternohyoid muscles (LNSS) have received attention. This article reports two cases of SSLN and LNSS recurrence and emphasizes the need for a thorough evaluation and consideration of the possibility of recurrence in this region. The clinical significance of the prophylactic dissection of SSLN and LNSS remains unclear, and further studies are required to determine its value. Regular follow-up checks of suspicious lymph nodes at SSLN and LNSS, as well as the central and lateral compartments, are recommended after thyroidectomy to detect recurrences and ensure appropriate management.

## Introduction

The rate of pathologically proven lymph node metastasis in papillary thyroid carcinoma (PTC) can reach up to 80%–90%, especially in the central compartment (1–3). Although most patients with node-positive PTC have a good prognosis with low mortality rates, the presence of node metastasis is known to increase the risk of locoregional recurrence and hence requires reoperation (4). Therapeutic neck dissection is recommended for patients with clinically node-positive PTC in the central and lateral compartments (5). Prophylactic central neck dissection (CND) remains controversial, although it is not usually recommended (6, 7).

Central compartment neck dissection incorporates levels VI and VII, consisting of the region bounded superiorly by the hyoid bone, laterally by the carotid arteries, anteriorly by the investing layer of the deep cervical fascia, posteriorly by the prevertebral layer of the deep cervical fascia, and inferiorly by the innominate artery. However, in the central neck area, there are lymph nodes at the suprasternal space (SSLN) and between the sternocleidomastoid and sternohyoid muscles (LNSS).

Preoperative evaluation of the SSLN and LNSS is usually overlooked and is not commonly considered for dissection, especially because this space does not fall under the general classification of the central or lateral compartments of the neck (8).

Lymph node metastases to SSLN and LNSS are generally rare in PTC, and they occur occasionally in patients with PTC with lateral compartment lymph node metastases, especially metastases at levels III and IV (9–11). Recurrences do occur at SSLN and LNSS despite the very low rate of incidence.

We encountered two cases of lymph node recurrence at the SSLN and LNSS in PTC, including one case of recurrence at the SSLN and one case at the LNSS. Herein, we report two cases of recurrence in this region and discuss its clinical significance. Written informed consent was obtained from the patients for publication.

## Case profiles

**Figure 1:**
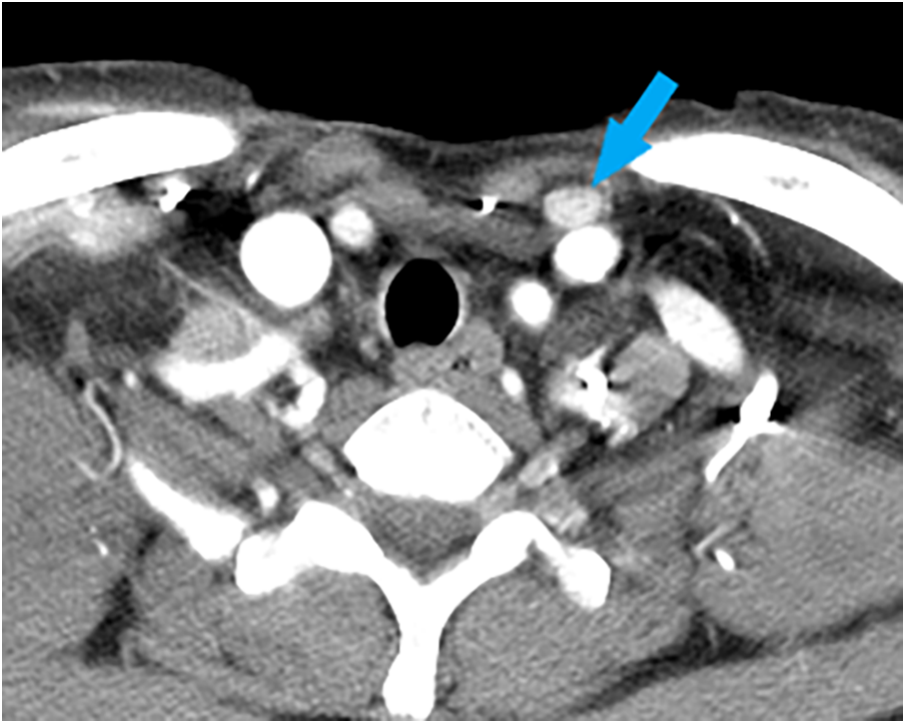
A computed tomography image of a metastatic lymph node (blue arrow), measuring 1.2 cm **×** 0.8 cm in size, at the left suprasternal space (SSLN).

**Figure 2:**
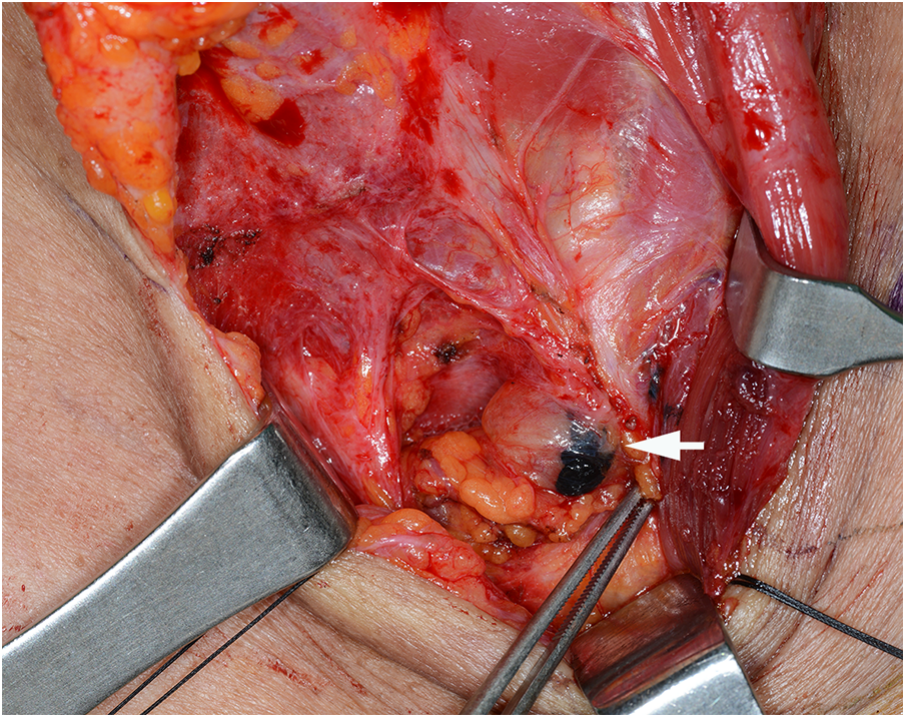
Surgical view of the metastatic lymph node, after tattooing (white arrow), at the left suprasternal space (SSLN).

Case 1: A 62-year-old female was diagnosed with PTC arising in the left thyroid lobe and a thyroglossal duct cyst by fine-needle aspiration cytology (FNAC) and imaging studies, such as ultrasonography (US) and computed tomography (CT). US and CT examinations revealed two suspicious thyroid nodules, 8 and 7 mm in size, in the mid-portion of the left lobe and a 2-cm thyroglossal duct cyst, without any suspicious nodes in the central or lateral compartments. The patient underwent total thyroidectomy and prophylactic CND along with a Sistrunk operation for a suspected PTC of a thyroglossal duct cyst. The final pathological examination revealed PTCs in the left thyroid lobe and thyroglossal duct cyst. In addition, an occult metastatic node was identified in the central compartment. The patient underwent radioactive iodine (RAI) ablation (150 mCi of ^131^I) 1 month postoperatively. There was no evidence of regional or distant metastasis or remnant thyroid tissue in the thyroid bed on the follow-up RAI scan. The postoperative course was uneventful, and there has been no recurrence for up to 5 years after surgery. However, routine check-up US and CT revealed a suspicious lymph node in the suprasternal space 5 years postoperatively (Figure 1), and FNAC confirmed metastatic PTC. The patient underwent selective neck dissection (SND), including SSLN and LNSS and levels III–V (Figure 2). The final pathology confirmed metastatic PTC in one lymph node of the suprasternal space, whereas 48 lymph nodes from levels III–V showed no metastasis. Eleven years after the revision surgery, the patient is doing well without recurrence.

**Figure 3:**
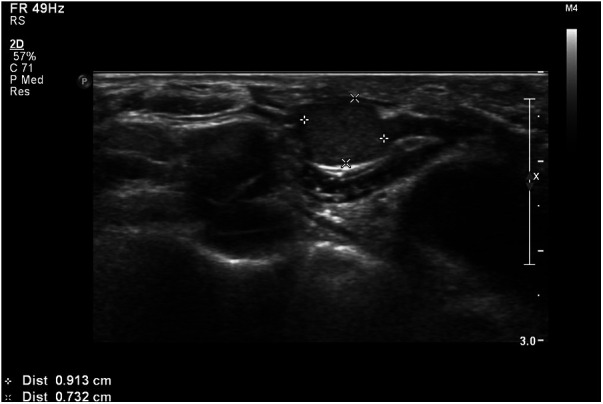
An ultrasound transverse view of a metastatic hypoechoic nodule, measuring 0.9 cm **×** 1.2 cm in size, within a lymph node between the right sternocleidomastoid and sternohyoid muscles (LNSS).

**Figure 4:**
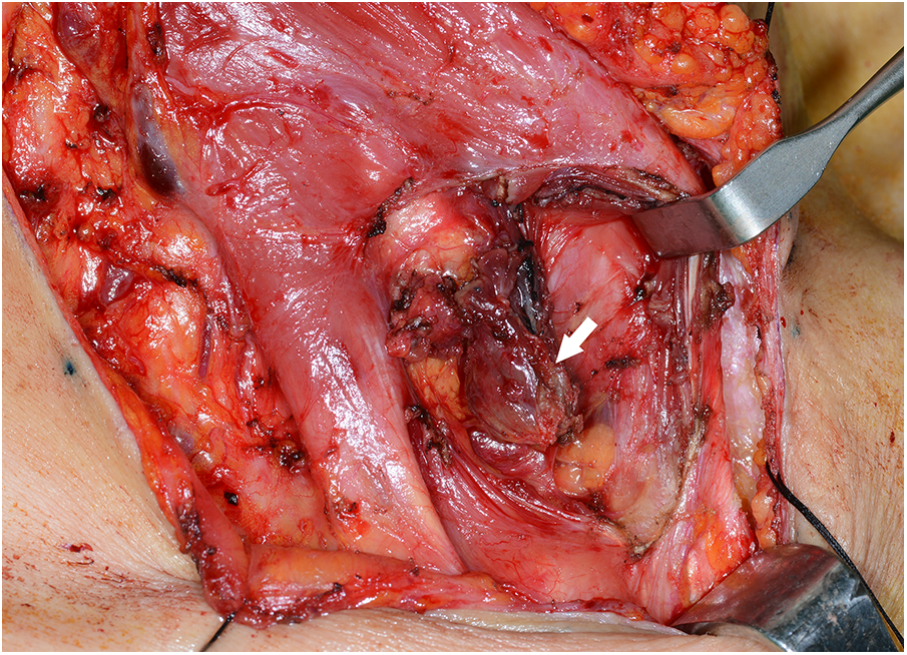
Surgical view of the metastatic lymph node (white arrow) between the right sternocleidomastoid and sternohyoid muscles (LNSS).

Case 2: A 58-year-old female was referred to our hospital for managing bilateral vocal cord palsy after thyroidectomy 6 years before she visited our hospital. According to her medical records, the patient had already undergone total thyroidectomy, CND, left SND including levels II–V, and postoperative RAI therapy (150 mCi of ^131^I). She also underwent a third reoperation on both thyroid beds: CND, SND of the right level IV, and left levels II–IV. In addition, the patient received a second round of RAI therapy (150 mCi of ^131^I). During her initial visit to our hospital, imaging revealed no definite evidence of local or regional lymph node recurrence. Posterior cordotomy was performed using a transoral CO_2_ laser for bilateral vocal cord palsy, and her airway problems improved. At the 1-year follow-up, 7 years after the initial surgery, a newly developed hypoechoic nodule, measuring 0.9 cm **×** 1.2 cm in size, was found in an LNSS (Figure 3), and FNAC proved metastatic PTC. The patient underwent neck dissection at the right SSLN and LNSS, right CND, and right SND at levels II–V (Figure 4). Pathology revealed three metastatic lymph nodes at LNSS. The patient underwent another round of RAI therapy (180 mCi of ^131^I). Subsequently, the patient was lost to follow-up after a 2-year disease-free period.

## Discussion

Several previous studies have attempted to define SSLN and LNSS in patients with PTC to determine their significance (10–13). Sun et al. initially defined lymph nodes in the area between the sternocleidomastoid and sternohyoid muscles, hence its name LNSS, bounded by the intersection of the sternocleidomastoid and sternohyoid muscles superiorly, the suprasternal fossa and clavicle inferiorly, and the sternohyoid muscle as its medial and lateral boundaries (10). The suprasternal space is composed of superficial and deep layers of the investing deep cervical fascia above the manubrium of the sternum, also known as the burn space (11). Lee et al. defined the superficial level VI, and the boundaries of superficial level VI are superior to the lower margin of the cricoid cartilage, inferior to the sternal notch and clavicle, lateral to the lateral border of the sternohyoid muscle, medial to the medial border of the sternohyoid muscle, anterior to the investing layer of the deep cervical fascia, and posterior to the sternohyoid muscle (8).

Although it has been suggested that SSLN and LNSS metastases may be associated with preoperative lateral lymph node metastasis, primary tumors located in the inferior pole, strap muscle invasion, extrathyroidal extension, and the strength of correlation vary considerably among studies (10–13). Sun et al. demonstrated an LNSS metastasis rate of 22.6% in patients with PTC and clinically positive lateral lymph nodes (10). Yu et al. reported an SSLN metastasis rate of 20.7%, with the primary tumor at the inferior pole, strap muscle invasion, and level IV metastasis as independent risk factors (11). Similarly, Kwon et al. showed a positive rate for SSLN to be 12.9% and a correlation between a primary site in the inferior pole and level IV metastasis (12).

Several routes of metastasis to superficial-level lymph nodes have been proposed. Sun et al. speculated that metastasis to the LNSS may result from an increasing tumor load after lateral neck metastasis or communication between the superficial or deep anterior cervical chain and the deep lateral cervical chain (10). Homma et al. suggested that fibro-fatty tissues, including metastases from level IV, may gradually spread into the suprasternal space due to the constant movement of the neck (9). Similarly, Kwon et al. suggested that metastatic lymph nodes from level IV migrate medially, and those from level VI migrate laterally into the suprasternal space. Despite these efforts, in PTC patients, general patterns of lymph node metastasis to SSLN and LNSS are still not completely understood, and controversies remain.

The risk of recurrence is substantially higher in patients with PTC with initial clinically apparent metastases to the cervical lymph nodes. While the reported overall rate of nodal recurrence in patients with PTC ranges from 1% to 6%, the risk of recurrence in clinical N0 and N1 necks showed significant differences, with average rates of 4% and 22%, respectively (14). Moreover, nodal recurrence in patients with PTC preferentially occurs in the ipsilateral mid-lower jugular nodes, while posterior triangle and skipped metastases are uncommon (15). Although speculated to be even rarer, the incidence of recurrence at SSLN and LNSS is unknown because the lymph nodes of this area have received attention only recently.

In the two cases reported here, SSLN and LNSS recurrences were discovered on routine postoperative follow-up US or CT scans. There were no suspicious lymph nodes at SSLN and LNSS before the primary thyroidectomy. Therefore, after the initial thyroidectomy, it is essential to perform regular follow-ups for any suspicious lymph nodes at SSLN and LNSS and in the central and lateral compartments.

It is unclear whether prophylactic dissection of SSLN and LNSS can prevent recurrence in this area. However, we disagree with prophylactic dissection of SSLN and LNSS because the rate of occult metastasis is low. No clear evidence for the benefits of prophylactic dissection of SSLN and LNSS has been established. Lee et al. advised against prophylactic dissection of superficial level VI lymph nodes, including SSLN and LNSS, in the absence of suspicious findings on preoperative imaging, based on the low rate (3.1%) of superficial level VI occult metastases in their study (8). Yuan et al. suggested that greater attention should be paid to the lymph nodes between the investing layer of cervical fascia and deep fascia of infrahyoid strap muscles in a selected number of PTC patients based on the presence of suspected metastasis (13). Dissection of SSLN and LNSS should be carefully considered in cases with suspicious lymph nodes or confirmed metastasis in primary or recurrent cases. Further prospective studies with more cases and longer follow-ups are necessary to determine the predictive value and clinical significance of SSLN and LNSS metastasis.

## Conclusion

Lymph node recurrence at the superficial level VI, including the SSLN and LNSS, can occasionally occur in patients with PTC. Therefore, careful evaluation and consideration should be given to the possibility of lymph node recurrence in this region.

## Data Availability

The raw data supporting the conclusions of this article will be made available by the authors, without undue reservation.
